# Cancer Care: Supporting Underserved and Financially Burdened Family Caregivers

**Published:** 2017-07-01

**Authors:** Betty R. Ferrell, Kate Kravitz

**Affiliations:** Division of Nursing Research and Education, City of Hope Comprehensive Cancer Center, Duarte, California

## Abstract

Family caregivers who provide care for a loved one with advanced cancer suffer physically and psychologically from the care demands of a family member with high symptom burden and a poor prognosis. Often, family members are also faced with financial burden and suffer financial strain from their loved one’s care demands. This article describes an ongoing test of a palliative care intervention to support financially burdened caregivers of family members who have advanced cancer. The intervention is designed to decrease family caregiver burden, increase skills preparedness, improve family caregiver quality of life, decrease psychological distress, and increase family caregiver self-care. This intervention is an individualized intervention customized to a particular caregiver and situation. It combines adult teaching principles, the National Comprehensive Cancer Network (NCCN) Distress Guidelines, the Institute of Medicine (IOM) Report on Cancer Care for the Whole Patient, the National Consensus Project (NCP) Clinical Practice Guidelines for Quality Palliative Care, and the concept of self-care. Initial findings indicate that financially strained family caregivers of family members with advanced cancer can benefit from self-care strategies that are designed to meet specific goals and individual needs when combined with a care plan and subsequent evaluations. However, findings indicate that financially strained caregivers may have limited resources and opportunities to utilize self-care strategies.

Health-care policies in the United States now mandate earlier hospital discharge following surgery and treatment. For oncology patients, family caregiving is the primary source of care. Not only are family caregivers central to the family member’s diagnosis and treatment, but also to survivorship and palliative care (Francis, Bowman, Kypriotakis, & Rose, 2011). Caregiving impacts caregivers physically and psychologically, especially when their loved one experiences many symptoms and a poor prognosis (Girgis, Lambert, Johnson, Waller, & Currow, 2013; van Ryn et al., 2011).

This article describes work in progress to test a palliative care intervention to support family caregivers who are financially strained and whose family member has advanced cancer. Related literature is reviewed, followed by a case example and suggestions for advanced practitioners so they may provide care to these families.

## IMPACT ON CAREGIVERS

Caregivers often suffer distress when providing care, a distress that differs from other emotional responses, such as depression or anxiety (Northouse, Williams, Given, & McCorkle, 2012). This distress includes an objective burden, the perceived disruption of elements of the caregiver’s life, and a subjective burden, when the caregiver finds the care demands to be excessive, causing an emotional impact (Savundranayagam, Montgomery, & Kosloski, 2011a). Patient, caregiver, and care situation characteristics also affect the caregiver.

Diagnosis, treatment, advanced disease stage, and the number of care tasks are patient characteristics. Caregivers of patients with advanced or terminal disease face greater demands than caregivers of patients still receiving disease-oriented treatments. Age, troubled relationships, absence of social support, along with feelings of guilt, inadequacy, loss of control, and a perception that the patient has unmet needs are typical caregiver characteristics (Savundranayagam, Montgomery, Kosloski, & Little, 2011b). Care environment characteristics include the type of care tasks, such as intimate body care, and intensely and tightly scheduled care. When caregivers report that caring for their loved one has caused at least one severe burden, and that they have suffered major life changes and cannot function normally, caregiving strain is considered high (Fletcher, Miaskowski, Given, & Schumacher, 2012; Savundranayagam, Montgomery, Kosloski, & Little, 2011b).

The cancer caregiver role has changed greatly in recent years. Previously, the caregiver provided care for the loved one in convalescence. Today, that role entails providing complex physical and emotional hands-on care in a home-based setting. Caregivers often misunderstand what this care will entail and the extent of responsibility and the impact it will have on their lives (Given, Given, & Sherwood, 2012). Families assume an intense role after initial diagnosis and are often thrust into caregiving following major surgeries, chemotherapy, and radiation therapy.

The extent of caregiver tasks may grow over time and at specific points in the illness journey, as care plans change. Caregivers must learn to manage symptoms while maintaining records, dispensing medications, and providing hands-on care, including managing catheters, injections, and infusion devices (Given, Given, & Sherwood, 2012). Caregivers also function as companions and aides; rendering emotional support, providing personal care, preparing meals, and offering transportation. Caregivers may also be faced with legal, medical, and financial tasks, including decisions on treatment, care goals, advance directives, home-care staffing, care transitions, death preparation, and funeral arrangements (Fletcher et al., 2012; Given et al., 2012; Savundranayagam et al., 2011b).

Cancer caregiver tasks are multifaceted, with little support from the current health-care system. Symptoms such as fatigue, pain, and dyspnea are common in patients with advanced cancer, and the caregiver may be faced with managing these distressing symptoms while also assisting with nutritional needs related to cachexia or nausea and significant functional decline. Patient depression and anxiety often accompany cancer, creating emotional burdens for the caregiver. The caregiver must face the reality that cancer frequently recurs and often ends in death. Virtually all areas of caregiving are impacted by the financial status of the patient and family (Sun et al., 2015b). Despite these negative impacts, there are caregiving benefits. Caregiver satisfaction may result from helping patients survive in early-stage disease and providing comfort and support in late-stage disease (Sun et al., 2015a).

**Quality of Life and Psychological Distress**

Caring for a loved one with cancer can impact the caregiver’s well-being. Although the experience of caregiving can be positive and evidence of a deep and fulfilling relationship, the caregiver and his or her quality of life (QOL) can suffer physically, psychologically, socially, and spiritually as a result of the caregiving. 

Cancer caregiving includes significant physical strain. As the patient’s disease and treatment-related symptoms increase, the cancer caregiver suffers caregiving-related symptoms, and physical well-being decreases (Goren, Gilloteau, Lees, & DiBonaventura, 2014). Cancer symptoms often require 24 hour/day attention, disrupting the caregiver’s sleep, causing fatigue, and increasing the caregiver’s mortality risk (Steele & Davies, 2014).

Research findings suggest that caregiving impacts the caregiver psychologically and may cause psychological distress, anxiety, and depression—stresses similar to those suffered by patients with declined functioning, as their terminal disease progresses. Some studies have shown that throughout the various illness stages, the caregiver may find the cancer experience more stressful than the patient (Sun et al., 2015a, 2015b).

Caregiving social demands arise from relationships and financial factors. The majority of caregivers are spouses, followed by adult daughters or daughters-in-law, and others include friends or extended-family members. Cancer can strain marital relationships, with depression on the part of the patient or spouse negatively impacting the relationship. Different communication styles may impact negatively on the patient’s and caregiver’s ability to cope (Wittenberg-Lyles, Goldsmith, Ferrell, & Ragan, 2013). Inadequate family communication can impair family functioning, with cancer and treatment costs exacerbating family difficulties and increasing caregiver burden. Couples may experience denial, avoidance, and conflict when coping with cancer, which in turn may harm their communication and support of one another (Wittenberg- Lyles et al., 2013). Patients and their spouses have reported communication difficulties with regard to cancer symptoms, prognosis, and emotional response to the disease.

The Clinical Practice Guidelines for Quality Palliative Care established by the National Consensus Project (NCP) suggest that patient and family meetings be held routinely with the health-care team (National Consensus Project for Quality Palliative Care, 2013). The meetings are recommended as a means to improve communication and create individualized care plans (National Consensus Project for Quality Palliative Care, 2013). The NCP guidelines recognize family caregivers and family issues as fundamental in palliative care and as part of the social well-being domain, throughout serious illness, not only at the end of life. Family meetings can also offer an opportunity to assess financial burden.

Spiritual well-being is important for cancer caregivers and their loved ones. Research suggests that caregivers find meaning in the cancer experience, just as cancer patients do (Sun et al., 2015b). In caregivers of patients who are long-term survivors, cancer can be a transformative experience, as they reprioritize their lives and find meaning in caregiving.

**Skill Preparedness**

Many caregiver tasks presuppose that necessary skills already exist, whether it be handling insurance claims and reimbursements, following medical instructions, utilizing devices such as catheters and home infusions (Steele & Davies, 2014), or caring for a patient physically: lifting, assisting with ambulation and nutrition, or managing pain and dyspnea treatments. Caregivers receive minimal support and little to no training or assessment of skill level.

## COST OF CANCER CAREGIVING

It is important for clinicians to assess financial concerns as part of the initial admission. This can provide for early referral to support services, legal assistance and social workers, or other service specialists who can help patients and caregivers be aware of all available support.

According to the American Society of Clinical Oncology (ASCO), "The State of Cancer Care in America 2016," by 2020, cancer-related costs may reach as high as $173 billion. Cancer care costs are rising more rapidly than other medical sector costs (ASCO, 2016). Cost reasons include the development of new treatments, increasing drug prices, and the sheer number of new patients and survivors. In 2015, more than 1.7 million new diagnoses were expected, and the number of US cancer survivors from 2014 to 2024 is expected to grow from 14.5 million to 19 million (ASCO, 2016).

Cancer care can take a significant financial toll on patients and their families. Out-of-pocket spending is becoming an increasing problem for patients and families. Zafar and colleagues have researched the effect of physicians’ failure to disclose the cost of treatments, which may be "financially toxic," likely to cause considerable financial strain, and impair patients’ well-being (Ubel, Abernethy, & Zafar, 2013; Zafar, 2016; Zafar et al., 2013). Financial burden does not end with treatment completion; cancer patients and their families continue to pay higher medical costs posttreatment than individuals without a cancer diagnosis (ASCO, 2016).

Virtually all indirect cancer care costs are the responsibility of patients and families. They include lost wages, secondary to unemployment and reduced hours of work. Some cancer patients may be too debilitated by treatments to return to full-time work, and family caregivers are forced to reduce working hours or quit completely due to caregiving responsibilities. Indirect costs include transportation and child care expenses. Family caregivers transport loved ones to and from treatments/visits, and families living far away from treatment centers must pay out-of-pocket costs for lodging and food. Caregivers with young children must secure and pay for child care. Although direct health-care costs may seem more substantial, indirect costs are a harsh reality for patients and caregivers on a daily basis, threatening the most basic needs and QOL. Studies that detail costs of caregiving are limited.

## INTERVENTIONS FOR CAREGIVERS

Previous research has centered on caregivers’ health problems created by caregiving demands, without consideration of the role self-care can play. The importance of caregivers’ caring for themselves, however, directly affects their ability to care for their loved one. Self-care includes rest, good nutrition, exercise, seeking counseling and support, routine health maintenance, and not ignoring one’s own serious illnesses or health conditions (Sun et al., 2015a, 2015b).

Few studies have tested cancer caregiver self-care strategies. Exercise, relaxation, and meditation, previously recommended for nurses and other health-care professionals, may also be useful for family caregivers, if individually tailored and with subsequent evaluation. However, self-care opportunities are financially dependent, and caregivers in financially strained circumstances may have limited resources and opportunities.

## FINANCIALLY BURDENED CAREGIVER STUDY

This study builds upon existing literature, applying descriptive data to test an intervention in a group of caregivers with loved ones diagnosed with a variety of cancer types, so study findings can be generalized across diagnoses. The caregiver is defined as the person identified by the patient as most involved in his/her care, whether or not the person is related. 

The framework for the Family Caregiver Palliative Care Intervention (FCPCI) combines adult teaching principles, the National Comprehensive Cancer Network (NCCN) Distress Guidelines (NCCN, 2003), Institute of Medicine (IOM) Report on Cancer Care for the Whole Patient (IOM, 2008), the NCP Clinical Practice Guidelines for Quality Palliative Care (National Consensus Project for Quality Palliative Care, 2013), and the concept of self-care. These concepts, along with the investigators’ preliminary studies on oncology patient caregivers, provide the basis for creating the content and methods for the tailored teaching of the FCPCI (Figure).

**Figure 1:**
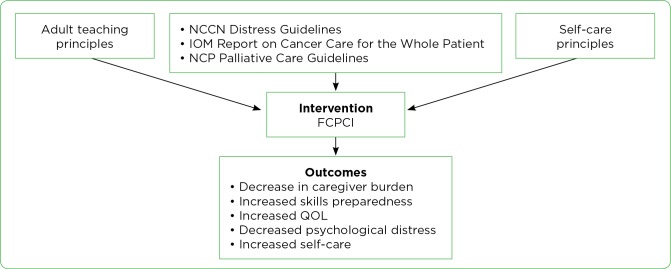
Family Caregiver Palliative Care Intervention (FCPCI) Framework. IOM = Institute of Medicine; NCCN = National Comprehensive Cancer Network; NCP = National Consensus Project for Quality Palliative Care; QOL = quality of life.

The adult teaching principles address specific adult characteristics and individual preferences and goals. The opposite of this model is an approach in which the same content and method of education is given to all. The FCPCI is an "individualized intervention" customized to a particular caregiver and situation.

The NCCN Guidelines on Distress Management (2003) provide Standards of Care for Distress Management, where patients, families, and health-care teams are informed that distress management is an integral part of care and that psychosocial services should be provided in the hospital and in the community. The use of the Distress Thermometer is one approach to assessing patient and family caregiver distress.

The IOM Report on Cancer Care for the Whole Patient: Meeting Psychosocial Health Needs (2008) defines "psychosocial care" as the psychological and social services and interventions that enable patients, their families, and health-care providers to optimize biomedical health care and to manage the psychological/behavioral and social aspects of illness and its consequences to better health. Family caregiver support is identified as the first line of defense for most cancer patients, and failure to address patients’ and caregivers’ psychosocial health needs is a key barrier to effective cancer patient treatment.

The FCPCI self-care component is based on the need for interventions to prevent caregiver health-related problems. Caregivers do not get enough rest and exercise, often have poor eating habits, ignore their own comorbidities, and have a decreased ability to cope with caregiving stresses as demands increase. Goal-oriented and tailored interventions are needed, especially to address financial constraints. As the model indicates, the FCPCI is hypothesized to decrease family caregiver burden, increase skills preparedness, improve family caregiver QOL, decrease psychological distress, and increase self-care. This model guides the study aims and hypotheses. To date, the study has accrued 130 of the planned 200 caregivers.

The intervention consists of four components addressing the QOL domains of physical, psychological, social, and spiritual well-being. The caregiver is given written information, and the content is taught over two to four sessions depending on the caregiver preference. Teaching sessions occur in person or by phone. Each session addresses the role of the family caregiver in meeting the patient’s needs in each domain, as well as in meeting their own needs.

## CASE STUDY

A caregiver, Julia, is the only daughter of a patient, Marilyn, who has stage IV lung cancer. Julia has a significant other, Sam, and one child from another relationship. Sam is very involved in supporting Julia and in co-parenting her daughter. They live with her mother and father due to the financial strain of the illness. Sam did not expect these changes in their new relationship, but he is trying to adapt to living with Julia’s parents.

Julia is very close to her mother. They have a very open and direct communication style. Julia states that she is "honored to care for her." Julia and Marilyn are aware of the poor prognosis, and Julia is committed to making sure her mother’s final days are safe, comfortable, and peaceful.

Julia’s greatest current concern is focused on plans for surgical palliation of the tumor, with the goal of reducing her mother’s suffering. She is also distressed about the precarious financial situation of the entire family. Marilyn receives disability, and her husband also receives disability; however, this income does not cover the basic costs of living. Julia works a full-time job, but there has been a change by her employer, and she is now concerned she may lose her job. Their landlord has expressed a desire to raise their rent but has agreed to wait until Marilyn is more stable. No one in the family was prepared for the extreme out-of-pocket costs of cancer.

## CASE DISCUSSION AND CONCLUSION

This case illustrates the many complexities of families facing the financial burden of cancer. Financial burdens can add to the physical, psychological, social, and spiritual caregiver experiences. Caregivers must balance their own relationships, such as in this case where Julia is a mother, partner to Sam, and daughter caring for her mother while also maintaining her employment. Caregivers such as Julia struggle with financial uncertainty, as they don’t know what care may be required as the illness progresses.

Table 1 is a guide for advanced practitioners to assess the many financial factors that may be impacting patients and families. The issues in Table 1 are derived from the families who have participated in the study thus far. Table 2 illustrates the costs of cancer caregiving and how they add to the financial burdens of a family.

**Table 1:**
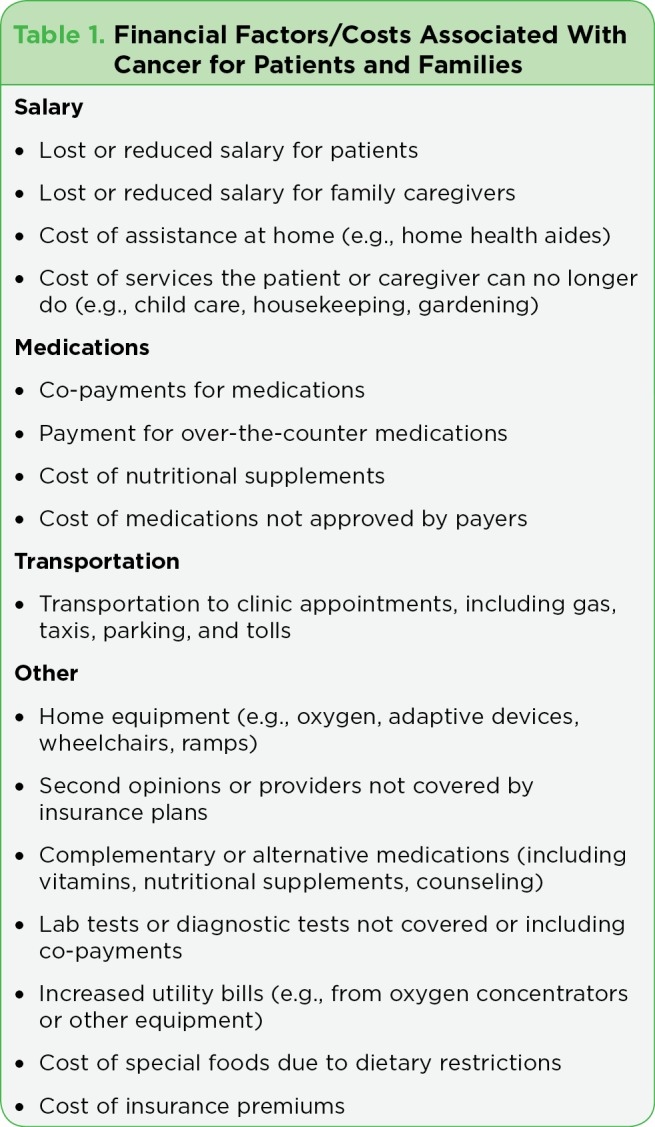
Financial Factors/Costs Associated With Cancer for Patients and Families

**Table 2:**
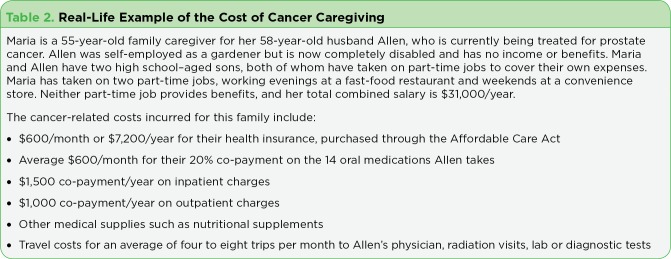
Real-Life Example of the Cost of Cancer Caregiving
